# Trends in camel research in South Asia: A bibliometric approach

**DOI:** 10.14202/vetworld.2024.2763-2773

**Published:** 2024-12-13

**Authors:** Mahmoud Kandeel

**Affiliations:** 1 Department of Biomedical Sciences, College of Veterinary Medicine, King Faisal University, Al-Ahsa 31982, Saudi Arabia; 2 Department of Pharmacology, Faculty of Veterinary Medicine, Kafrelsheikh University, Kafrelsheikh 33516, Egypt

**Keywords:** bibliometric analysis, camel research, publication trends, South Asia

## Abstract

**Background and Aim:**

Camels play a crucial role in South Asia’s cultural, economic, and ecological landscape. This study aimed to conduct a systematic bibliometric analysis of camel research from South Asia. This study sought to provide an overview of the topic’s development and current and evolving themes by examining publication patterns, citation metrics, authorship trends, and thematic evolutions.

**Materials and Methods:**

The data for this study were obtained from the Scopus database. Bibliometric analysis was conducted using VOSviewer and the Bibliometrix package in R Studio to analyze publication trends, author productivity, collaboration patterns, journal impacts, keyword analyses, institutional contributions, and research outputs from individual countries.

**Results:**

The bibliometric analysis of camel research in South Asia identified 1106 documents from 320 sources involving 2443 authors, with an international coauthorship rate of 16.91%. The primary clusters of research topics were camel biology and the veterinary sciences, focusing on the biological and health aspects of camels; camel milk and its products, emphasizing the role of camel milk in human nutrition and health; the genetics and molecular biology of camels; the pathogens and diseases of camels and their control; camel tissues and structures; and the effects of camel products on human health. India and Pakistan are the leading sources of such articles. Trending and evolving topics in camel research in South Asia continue to increase, forming a dynamic landscape. Key themes include growing interest in camel products’ nutritional and therapeutic properties, particularly camel milk and bioactive compounds. The antioxidant and anti-inflammatory properties of camel products and their bioactive peptides have also gained research attention. The evolution from traditional studies of camel physiology and disease to molecular and genomic research underscores a shift toward a more detailed, mechanistic understanding of camel biology and health.

**Conclusions:**

This bibliometric study highlighted the significant growth and diversification of camel research in South Asia over the past decades. The study emphasized the need for continued support and collaboration to gain knowledge of the unique attributes of camels and their regional and global benefits.

## Introduction

Camels have been crucial in South Asia’s cultural, economic, and ecological landscape [1, 2]. These resilient animals, which adapt to harsh arid and semi-arid environments, have served as vital resources for transportation, food, fiber, and labor across the region [3]. In recent decades, the importance of camels has grown, with increasing recognition of their potential in sustainable agriculture [4].

The South Asian region has diverse habitats and socioeconomic contexts [5]. Camels are still integral to local livelihoods and ecosystems in the region [6]. As climate change and desertification pose increasing challenges to agriculture and food security in parts of South Asia, the adaptability and resilience of camels have sparked renewed scientific interest in them [7, 8]. Research on camels in South Asia spans various disciplines, including veterinary science, genetics, reproductive biology, nutrition, pharmacology, and socioeconomic conditions [9]. In recent years, there has been a surge in publications exploring the potential of camel milk as a functional food, the use of camel-derived products in traditional and modern medicine, and the genetic characterization of various camel breeds native to the region [10, 11]. In addition, there is growing interest in the role of camels in sustainable land management and their potential to contribute to climate change mitigation strategies [12].

Despite the obvious increase in camel-related research from South Asia, no comprehensive analysis of the trends, patterns, and impacts of the publications that feature this research has been conducted. This study aimed to conduct a systematic bibliometric analysis of camel research from South Asia from 1921 to 2024. Also, to provide a comprehensive overview of the topic’s development and current status by examining publication patterns, citation metrics, authorship trends, and thematic evolution.

## Materials and Methods

### Ethics approval

This study was based on the extraction and analysis of the published articles so, ethical approval was not necessary.

### Study period and location

The study started in August 2024 and the data was updated on 3^rd^ October 2024. The study was carried out in King Faisal University, Saudi Arabia.

### Data sources and search strategy

The data used in this study were obtained from the Scopus database, which was selected for its comprehensive coverage of scientific literature. The search query was designed to capture all relevant publications featuring South Asian camel research. The countries searched were Bangladesh, Bhutan, India, Maldives, Nepal, Pakistan, Sri Lanka, and Afghanistan.

The search string used was TITLE (camel) AND (LIMIT-TO (AFFILCOUNTRY, “Afghanistan”) OR LIMIT-TO (AFFILCOUNTRY, “India”) OR LIMIT-TO (AFFILCOUNTRY, “Nepal”) OR LIMIT-TO (AFFILCOUNTRY, “Pakistan”) OR LIMIT-TO (AFFILCOUNTRY, “Sri Lanka”) OR LIMIT-TO (AFFILCOUNTRY, “Bangladesh”). This search was conducted on October 3, 2024, and yielded an initial dataset of 1106 records. The countries listed in the database that were sources of camel-related studies were Bangladesh, India, Nepal, Pakistan, Sri Lanka, and Afghanistan.

### Inclusion and exclusion criteria

The inclusion criteria for this bibliometric analysis were that the studies had to be indexed in the Scopus database, focus on camel research, and affiliated with South Asian institutions. The analysis included various types of publications, such as articles, reviews, conference papers, book chapters, and short surveys; there were no limits on the publication dates. The exclusion criteria stipulated that duplicate records, errata and corrections, and publications not directly related to camel research, such as studies in which the word camel was used metaphorically rather than literally, were not considered for this study.

### Data cleaning and preprocessing

The data were exported in CSV format from the Scopus database; data cleaning and preprocessing were performed using R Studio 2024.09.1 Build 394 (https://posit.co/downloads/). This process involved removing duplicate entries, standardizing author names and affiliations, harmonizing keywords by merging synonyms, and correcting inconsistencies in the bibliographic information.

### Bibliometric analysis

The bibliometric analysis was conducted using two primary tools. VOSviewer version 1.6.18 (Centre for Science and Technology Studies, Leiden University, The Netherlands, https://www.vosviewer.com/) was used to construct and visualize bibliometric networks, including coauthorship networks, keyword co-occurrence networks, and citation networks [13–15]. In addition, R Studio with the Bibliometrix package version 3.1.4 (K-Synth Srl, Academic Spin-Off of the University of Naples Federico II, https://www.bibliometrix.org/home/index.php) was employed for comprehensive bibliometric and scientometric analyses, including performance analysis, science mapping, and trend analysis [16].

The analysis focused on the key aspects of publication trends over time, author productivity and collaboration patterns, the impact and relevance of journals, keyword analysis to identify research hotspots, institutional contributions and collaborations, and research output and collaborations of the countries included.

### Analysis of publication trends

The publication trends were analyzed to understand the evolution of camel research in South Asia. This analysis included annual publications from 1921 to October 03, 2024, the cumulative growth of publications over time, the identification of key periods of growth or decline in research output, the analysis of document types (e.g., articles, reviews, conference papers), and the range of subject areas featured in publications.

Descriptive statistics, including central tendency and dispersion measures for various bibliometric indicators, were computed to provide an overview of the dataset. Time series analysis was performed to identify significant trends or patterns in research output over the years. The results of these analyses were visualized using plots and charts to effectively communicate the trends and patterns identified in the camel research landscape of South Asia.

## Results

### Dataset characteristics

**Figure-1:**
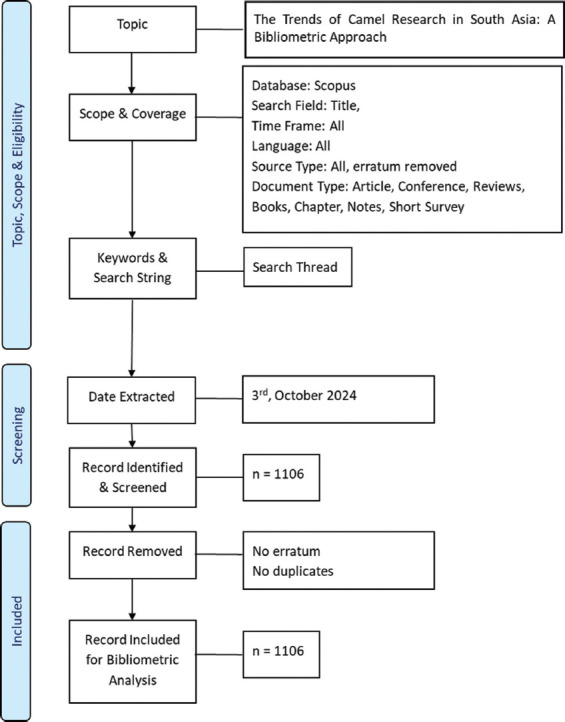
Preferred Reporting Items for Systematic Reviews and Meta-Analyses flow diagram showing the data retrieval and analysis processes.

Initially, 1106 records were identified in Scopus using the search keywords. After determining that there were no duplicates or errata among these articles, 1106 records were ultimately included in the bibliometric analysis. The data included authors’ names, unique identifiers (IDs), article titles, and bibliographic details such as the year of publication, source title, number of articles, volume number, issue number, and page number of each article. Citation metrics, DOIs, and links to full texts were also noted. Author affiliations and abstracts were included, along with keywords, funding details, references, and correspondence addresses. Other captured data included conference information, ISSN, ISBN, CODEN, PubMed ID, language, document type, publication stage, open access status, source, and EID. Figure-1 shows the Preferred Reporting Items for Systematic Reviews and Meta-Analyses flow diagram of our systematic approach to the bibliometric analysis of camel research trends in South Asia.

Data analysis identified 1106 documents from 320 sources, written by 2443 authors, with 4.79 co-authors per document. The international coauthorship rate was 16.91%.

### Publication trends

**Figure-2:**
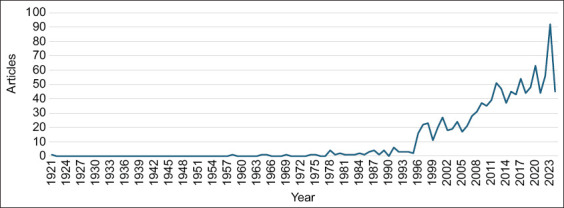
Annual number of scientific articles on camels in South Asia from 1921 to 2024.

The analysis revealed a significant growth in camel research in South Asia from 1921 to 2024, with a notable surge since 1996. The number of articles peaked in 2023 at 92 articles (Figure-2).

### Authors and co-authors by country

**Figure-3:**
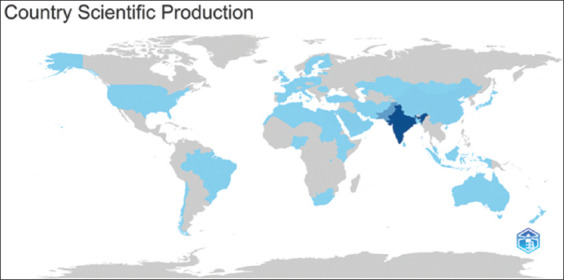
Authorship and co-authorship of camel research in South Asian countries. Countries in dark blue had the largest numbers, and countries in medium or light blue had smaller numbers.

The number of authors and co-authors of articles on camels in South Asia was greatest in India, reflecting the significant role played by India in camel research. Pakistan followed closely behind. Other notable countries included Saudi Arabia, China, Oman, the UAE, Egypt, and the United States (Figure-3). The presence of these countries was due to a fraction of 16.91% of collaborative work in the retrieved dataset.

### Countries of origin of the corresponding authors

A review of the corresponding country-specific articles revealed a prominent role for India and Pakistan in camel research. India led with 426 articles (38.51% of all the articles in the analysis), indicating its significant contribution to the topic. Pakistan followed with 155 articles (14.01% of all the articles), indicating strong engagement in camel-related studies.

**Figure-4:**
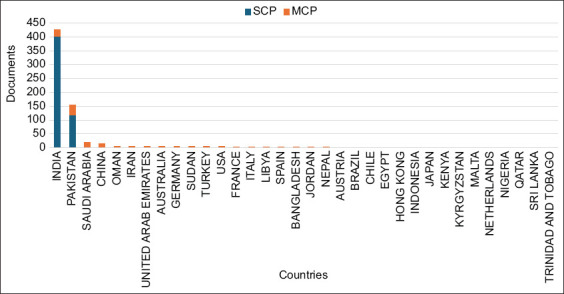
Countries of origin for corresponding camel research articles in South Asia.

Other countries showing coauthorship with South Asian countries, such as Saudi Arabia, China, Oman, Iran, and several European and Asian countries, contributed minimally to the research topic, indicating that their contribution was more of a niche or emerging interest (Figure-4).

### Authors who published the most articles

**Figure-5:**
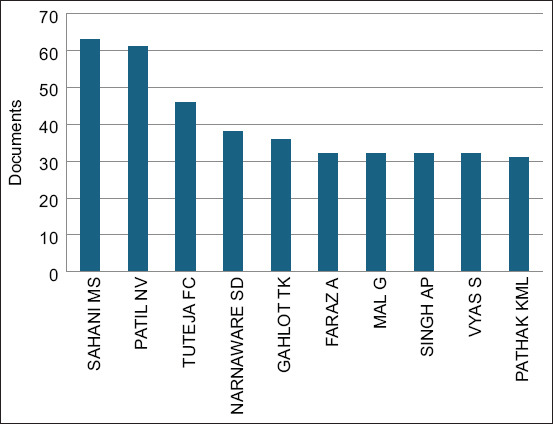
Authors of the highest number of camel research articles in South Asia.

Of the authors who authored or co-authored the included articles on camel research, we found that Sahani MS led with 63 articles (or 17.77% of the articles included), reflecting a substantial and influential presence in the field. Patil NV and Tuteja FC followed with 61 and 46 articles, respectively, representing significant research output. Narnaware SD and Gahlot TKA also contributed notably with 38 and 36 articles, respectively. Other key contributors included Faraz A, MAL G, Singh AP, Vyas S, and Pathak KML (Figure-5).

### Institutions with the most frequently published publications

**Figure-6:**
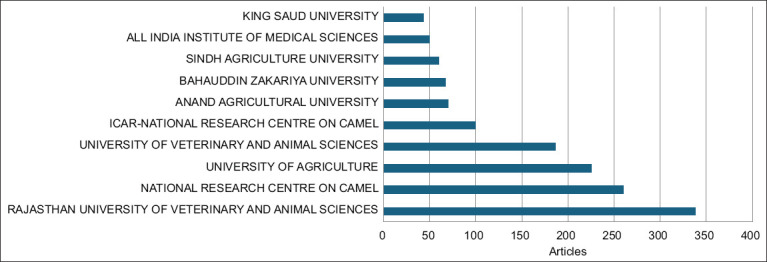
The 10 institutions producing the most camel-related articles in South Asia.

Figure-6 shows the key academic and research institutions contributing to camel-related research, with the Rajasthan University of Veterinary and Animal Sciences leading the list with 338 articles. The National Research Center on Camels (260 articles) and the University of Agriculture (225 articles) contributed significantly. Other notable institutions included the University of Veterinary and Animal Sciences (186 articles) and the Indian Council of Agricultural Research-National Research Center on Camels (100 articles). The remaining institutions, such as Anand Agricultural University and Bahauddin Zakariya University, contributed a smaller but notable number. King Saud University in Saudi Arabia has 43 publications. These publications indicate the prominent international collaboration of King Saud University with South Asian institutes in camel research.

### Core sources of camel-related research in South Asia

**Figure-7:**
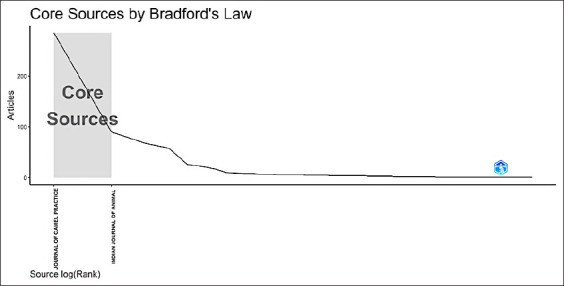
Core sources of camel research in South Asia, as identified by Bradford’s law.

Figure-7 illustrates how Bradford’s law was used to identify the core sources of camel-related research in South Asia. The steep decline in the number of articles as we moved away from core sources is evident. The shaded area labeled “core sources” highlights the journals that contributed the most articles to this research field, with the Journal of Camel Practice and the Indian Journal of Animal Science appearing as the top contributors.

### Influential journals in which camel-related articles were published

**Figure-8:**
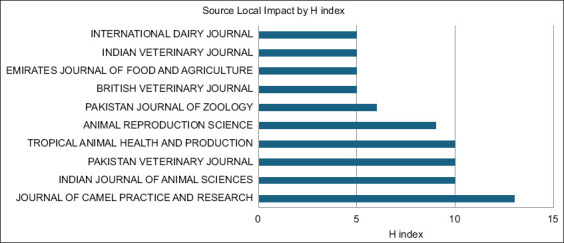
The 10 most influential journals publishing camel research in South Asia.

Journals publishing camel research reported varying impacts and longevity. Figure-8 lists the 10 most influential journals ranked by their h-indexes.

The Journal of Camel Practice and Research had an h-index of 13, indicating its strong influence and relevance. The following closely followed were the Indian Journal of Animal Sciences, Pakistan Veterinary Journal, and Tropical Animal Health and Production, each with an h-index of 10, reflecting their significant contributions and citations. Animal Reproduction Science had an h-index of 9. In contrast, other journals, such as the Pakistan Journal of Zoology and the British Veterinary Journal, had slightly lower h-indices of 6 and 5, respectively. Journals such as the Emirates Journal of Food and Agriculture, Indian Veterinary Journal, and International Dairy Journal also had an h-index of 5, showing that they had consistent scholarly output and moderate impacts in their respective domains.

### Keyword clusters

**Figure-9:**
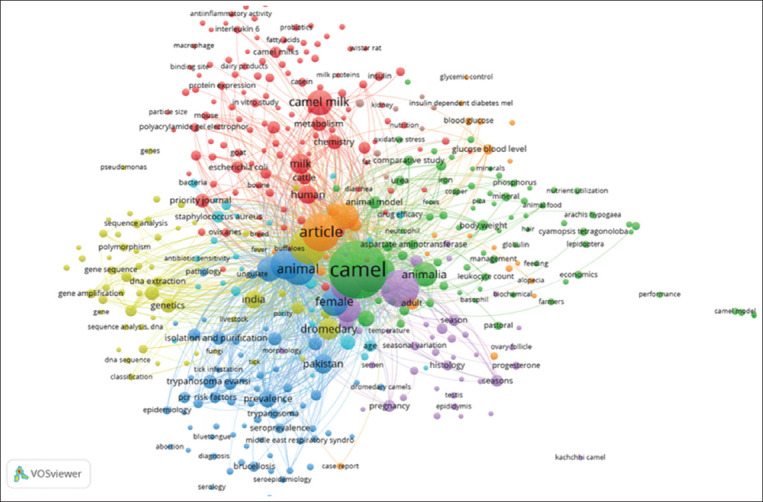
Clusters of keywords used in camel research in South Asia.

Six clusters of camel research topics in South Asia are listed in Figure-9. A green cluster shows camel biology and the role of the veterinary sciences in camel research. This cluster also centered on traits of dromedary camels. The key topics in this cluster were camel anatomy and biology, aspects of their health and reproduction, and veterinary care. Terms such as animalia, animal model, leukocyte count, drug efficacy, and the names of several minerals were important for gathering data on veterinary practices.

The red cluster shows the topics of camel milk and its components. The primary focus was on camel milk’s role in human nutrition, particularly its impact on metabolic health. Terms such as milk, casein, milk proteins, insulin, and chemistry were related to the nutritional value of camel milk and its potential health benefits, especially in managing conditions such as diabetes.

The yellow cluster in Figure-9 represents topics in genetics and molecular biology. The focus is on DNA sequencing, gene expression, and polymorphism. Researchers in these fields used terms such as genetics, sequence analysis, DNA extraction, and genomic DNA. They focused on genetic mapping, evolutionary studies, and the identification of molecular markers to understand the genetic foundations that influence camel traits, adaptations, and overall health.

The blue cluster in Figure-9 comprises the topics of pathogen and disease control. The research highlights were camel disease and epidemiology, particularly in regions such as Pakistan. This cluster was concerned with the prevalence and control of infectious diseases such as brucellosis, trypanosomiasis, and Middle East respiratory syndrome coronavirus and the seroprevalence of various pathogens.

The purple cluster in Figure 9 comprises topics related to camel issue and structures. The focus was on the physiology and histology of camels, particularly seasonal variations in camel tissues and structures and aspects of pregnancy and the reproductive system.

The orange cluster in Figure 9 shows the effects of camel product consumption on human health. Researchers used terms such as blood glucose, glucose level, oxidative stress, insulin-dependent diabetes, nutrition, and oxidative stress in their studies.

### Affiliations among institutions, article authors, and keyword networks used by authors

**Figure-10:**
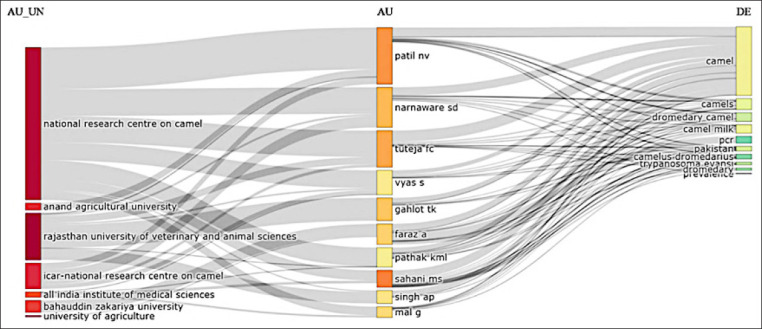
A three-field plot illustrating affiliations among institutions, authors, and keyword networks of camel research in South Asia.

Figure-10 shows a three-field plot illustrating affiliations among institutions (left), authors (center), and keywords (right) used by authors in camel research. The National Research Center on Camel (India) and the Rajasthan University of Veterinary and Animal Sciences were prominent in these affiliations and linked to leading authors such as Patil NV, Narnaware SD, Tuteja FC, Vyas S, Gahlot TK, Faraz A, Pathak KML, and Sahani MS. These authors studied key research topics such as camels, camel milk, dromedary camels, polymerase chain reaction in camels, and the occurrence of *Trypanosoma evansi* in camels. The dense network of links showcases the collaborations among institutions, researchers, and camel research topics. Figure-10 also highlights multiple affiliations and several output keywords for each author listed, indicating the broad interest in and diverse research work in the field.

### Affiliations among institutions, journals, and keyword networks used by the authors

**Figure-11:**
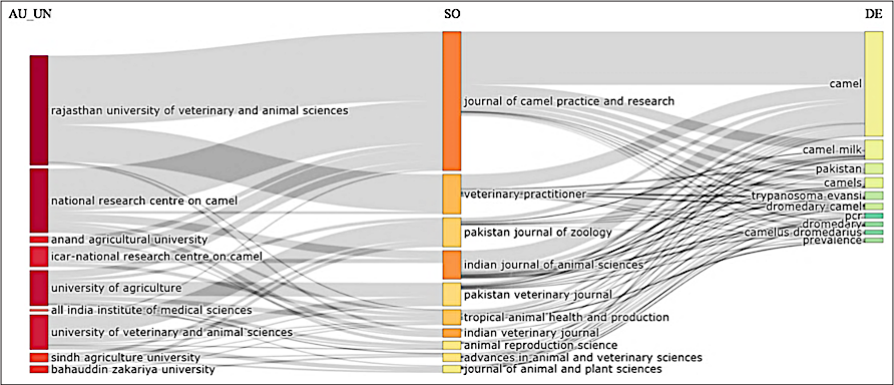
A three-field plot illustrating affiliations among institutions, journals, and keyword networks used by authors of camel research in South Asia.

Figure-11 displays a three-field plot highlighting the relationships among institutions (left), journals (center), and keywords used by authors (right). The National Research Center on Camel and the Rajasthan University of Veterinary and Animal Sciences are again prominent institutions, contributing significantly to journals such as the *Journal of Camel Practice and Research* and *Veterinary Practitioner*. These journals publish extensively on topics such as camels, camel milk, *T. evansi*, and *Camelus dromedarius*.

### Trending and evolving camel research topics in South Asia

**Figure-12:**
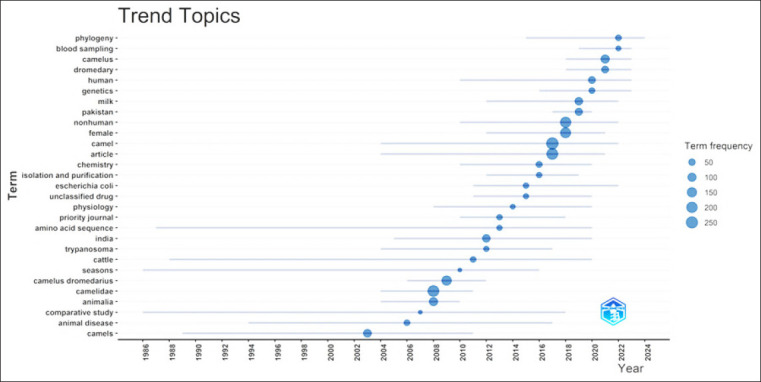
Trending and evolving camel research topics in South Asia.

Figure-12 illustrates the evolution of various research themes in camel studies from 1996 to 2024 in terms of their frequencies. The Y-axis lists terms used in specific research interests or topics, while the X-axis shows the timeline in years. The size of the circles correlated with the frequency of each term, indicating how often these topics appeared in the studies over time.

#### Classical topics

Terms such as animal diseases, Trypanosoma, and season appeared early in the timeline, indicating that these topics have long been of interest.

#### Emerging topics

Figure-12 highlights emerging areas in camel research, such as phylogeny, camel milk, and genetics, and shows a notable increase in the frequency of these topics in recent years. These topics reflect a growing interest in the health benefits of camel products, particularly in relation to their potential as bioactive compounds.

#### The most common trends

The most frequently occurring terms, such as comparative study, season, and amino acid sequence, dominate the research landscape, highlighting the central themes of camel studies.

## Discussion

This bibliometric analysis highlighted important camel research aspects in South Asia. Since 1996, there has been a notable surge in output. This increase likely reflects a growing recognition of camels’ importance in sustainable agriculture, food security, and climate change adaptation. The peak was in 2023, with 92 articles suggesting a high level of interest and funding in the field. This trend may also indicate a shift in research priorities to sustainable livestock practices and the unique adaptations of camels to arid environments [17]. The sparse early research followed by rapid growth could reflect the changing perceptions of camels from traditional livestock to subjects of scientific inquiry that might offer potential solutions to modern challenges.

The countries of origin of the publications underscore India’s leading role in camel research in South Asia while also highlighting substantial contributions from Pakistan and other countries [9, 18]. The broad regional and international interest, including significant input from countries such as Saudi Arabia, China, and others, reflects global academic and scientific cooperation in camel research [9].

The corresponding authors’ countries of origin analysis highlights India and Pakistan as having leading roles in camel research, with significant contributions also coming from Iraq and Saudi Arabia. The presence of multi-country publications (MCPs), particularly from Saudi Arabia, China, and the UAE, underscores the collaborative nature of camel research and its global scientific relevance. India leads with 426 articles (38.52% of the total research articles), most of which are single-country publications, indicating a focus on domestic research, with only 24 MCPs, representing 5.63% of the total articles. In contrast, Pakistan produced 155 articles (14.01% of the total), with a higher proportion of international collaborations, as shown by its 38 MCPs (24.52%). This suggests that while India dominated the number of articles, Pakistan placed greater emphasis on global collaborations in camel research.

The Rajasthan University of Veterinary and Animal Sciences led the academic research, indicating its strong focus on veterinary and camel studies. The National Research Center on Camel and the University of Agriculture also made significant contributions, highlighting their specialized focus on agriculture and animal sciences. The presence of King Saud University in Saudi Arabia added an international dimension to the predominantly Indian and Pakistani research landscape. These affiliations reflected concentrated efforts in South Asia, particularly in India and Pakistan, to conduct veterinary and agricultural research related to camels.

The application of Bradford’s law to identify core sources showed that the Journal of Camel Practice and the Indian Journal of Animal Science were the top contributors. These journals were the most productive in terms of number and thus played a critical role in disseminating camel research. As we moved further along the continuum of core sources, the number of articles contributed by other sources dropped sharply, confirming the law’s principle that a small number of core journals produce the majority of relevant articles, while many other sources contribute fewer publications.

Trending topics in camel research in South Asia have evolved significantly over the years. A key trend is the increasing focus on the nutritional and therapeutic properties of camel milk, particularly in managing metabolic disorders such as diabetes [19, 20]. The use of terms such as antioxidants [21], lactoferrin [22], and bioactive peptides [23] indicated a growing interest in the health benefits of camel products. Research has increasingly explored how the unique composition of camel milk, including its proteins and fatty acids, can offer solutions to modern health challenges, such as oxidative stress [24] and inflammation [25]. This shift underscores the move toward integrating camel products into functional food and nutraceutical industries, where their bioactive compounds can be studied for their preventive and therapeutic potential.

Another significant evolving trend is the shift from traditional camel health and disease studies to more advanced genomic and molecular biology research [26–28]. While earlier studies focused on camel physiology, diseases, and histopathology [29, 30], there is a growing emphasis on genetics, sequence analysis, and molecular markers [31, 32]. This shift suggests a deeper mechanistic exploration of camel biology to develop personalized veterinary care and breeding programs based on genetic insights. The rise in the use of polymerase chain reaction analysis, genomics, and DNA sequencing technologies also reflects broader trends in scientific research, as camels have become subjects of molecular and genetic studies aimed at improving health outcomes, breeding, and disease resilience.

This bibliometric analysis provides a comprehensive overview of camel research trends in South Asia, offering valuable insights into the field’s evolution, key contributors, and emerging themes. The study’s strengths lie in its extensive dataset, spanning over a century of research, and its multi-faceted approach to analyzing publication patterns, authorship trends, and research topics. However, limitations of the study include the reliance on a single database (Scopus), which may not have captured all relevant publications, particularly those in regional or non-indexed journals. In addition, the analysis did not account for the quality or impact of individual studies beyond their citation metrics. Future research could address these limitations by incorporating data from multiple databases, including qualitative assessments of research impacts, and exploring socioeconomic factors that drive research trends. Further investigation into the practical applications of camel research findings and their impacts on policy and industry practices is also valuable. Finally, a comparative analysis of camel research trends in other regions could provide a global perspective on the field’s development.

## Conclusion

This bibliometric study illuminates the significant growth and diversification of camel research in South Asia over the past decades. The analysis reveals a shift from traditional husbandry practices to more specialized scientific inquiries, reflecting the growing recognition of camels as potential solutions to contemporary challenges such as food security and climate change adaptation. The emergence of key research institutions, prolific authors, and influential journals has shaped camel science in the region. The diverse range of research topics, from milk properties to genetics and diseases, underscores the multidisciplinary nature of this field. As camel research continues to evolve, it holds promise for innovative solutions to regional and global challenges, emphasizing the need for continued support and collaboration in this important area.

## Data Availability

Supplementary data can be available from the corresponding author upon a reasonable request.

## Author’s Contributions

MK: Conceptualization, methodology, software, validation, formal analysis, investigation, resources, data curation, writing – original draft preparation, and writing – review and editing, The author has read and approved the final manuscript.
